# Biocompatibility and Osteogenic Activity of Samarium-Doped Hydroxyapatite—Biomimetic Nanoceramics for Bone Regeneration Applications

**DOI:** 10.3390/biomimetics9060309

**Published:** 2024-05-22

**Authors:** Mihaela Balas, Madalina Andreea Badea, Steluta Carmen Ciobanu, Florentina Piciu, Simona Liliana Iconaru, Anca Dinischiotu, Daniela Predoi

**Affiliations:** 1 Department of Biochemistry and Molecular Biology, Faculty of Biology, University of Bucharest, 91-95 Splaiul Independentei, 050095 Bucharest, Romania; mihaela.balas@bio.unibuc.ro (M.B.); madalina-andreea.badea@bio.unibuc.ro (M.A.B.); 2 National Institute of Materials Physics, No. 405A Atomistilor Street, 077125 Magurele, Romania; ciobanucs@gmail.com (S.C.C.); simonaiconaru@gmail.com (S.L.I.); dpredoi@gmail.com (D.P.); 3 Department of Anatomy, Animal Physiology and Biophysics, Faculty of Biology, University of Bucharest, 91-95 Splaiul Independentei, 050095 Bucharest, Romania; florentina.cojocaru@unibuc.ro

**Keywords:** hydroxyapatite, samarium, MC3T3-E1 preosteoblasts, bone regeneration, bone tissue engineering, biocompatibility evaluation, alkaline phosphatase, wound healing

## Abstract

In this study, we report on the development of hydroxyapatite (HAp) and samarium-doped hydroxyapatite (SmHAp) nanoparticles using a cost-effective method and their biological effects on a bone-derived cell line MC3T3-E1. The physicochemical and biological features of HAp and SmHAp nanoparticles are explored. The X-ray diffraction (XRD) studies revealed that no additional peaks were observed after the integration of samarium (Sm) ions into the HAp structure. Valuable information regarding the molecular structure and morphological features of nanoparticles were obtained by using Fourier-transform infrared spectroscopy (FTIR), transmission electron microscopy (TEM), and X-ray photoelectron spectroscopy (XPS). The elemental composition obtained by using energy-dispersive X-ray spectroscopy (EDS) confirmed the presence of the HAp constituent elements, Ca, O, and P, as well as the presence and uniform distribution of Sm^3+^ ions. Both HAp and SmHAp nanoparticles demonstrated biocompatibility at concentrations below 25 μg/mL and 50 μg/mL, respectively, for up to 72 h of exposure. Cell membrane integrity was preserved following treatment with concentrations up to 100 μg/mL HAp and 400 μg/mL SmHAp, confirming the role of Sm^3+^ ions in enhancing the cytocompatibility of HAp. Furthermore, our findings reveal a positive, albeit limited, effect of SmHAp nanoparticles on the actin dynamics, osteogenesis, and cell migration compared to HAp nanoparticles. Importantly, the biological results highlight the potential role of Sm^3+^ ions in maintaining cellular balance by mitigating disruptions in Ca^2+^ homeostasis induced by HAp nanoparticles. Therefore, our study represents a significant contribution to the safety assessment of both HAp and SmHAp nanoparticles for biomedical applications focused on bone regeneration.

## 1. Introduction

Loss of bone due to non-healing fractures, osteoporosis, and bone tumors constitutes a significant global health concern, contributing substantially to human disability worldwide [1,2,3,4].

A variety of natural or synthetic biomaterials, such as metals, ceramics, polymers, and their composites, are currently being developed or investigated as scaffolds or implants for bone grafting to promote bone regeneration and reconstruction [5]. Their therapeutic efficiency is often challenged by suboptimal biocompatibility, inadequate bone formation, as well as mechanical and biological characteristics mismatching those of native bone [6,7].

Hydroxyapatite (HAp), a calcium phosphate bioceramic with the chemical formula [Ca_10_(PO_4_)_6_(OH)_2_] and a Ca/P ratio of 1.67, stands as the foremost biomaterial in bone tissue engineering [8]. As the predominant inorganic constituent in human skeletal structures, which encompass both bone and dental tissues, HAp stands out for its superior attributes, including enhanced biocompatibility, bioactivity, and osteoconductivity when compared to alternative materials [9,10]. Notably, its microstructural attributes, especially the pore size, can be precisely manipulated. The surface reactivity of HAp depends on its sizes, forms, and functional groups [8]. The versatility of HAp underpins its extensive utility in a variety of orthopedic and dental applications, such as bone filling, coating materials for prosthetic metallic implants, bone tissue engineering scaffolds, substitute matrices for bone grafts, composites with antibacterial properties, drug delivery systems, bioimaging and diagnosis materials, and biosensors [8,11].

In the last decade, HAp with a nanoscale size termed nanohydroxyapatite (nanoHAp) has been proposed as a better candidate for bone tissue repair. This is largely due to its enhanced surface activity and ultrastructure. These characteristics contribute to improved densification and sinterability, which could potentially enhance fracture toughness and other mechanical properties [12,13].

This form of HAp demonstrates superior absorbability and biological activity, resulting in enhanced cellular responses compared to traditional HAp [14]. For example, previous investigations have established a relationship between HAp size, cell proliferation, and apoptosis. Notably, it was shown that nanoHAp (20 nm) was more effective than larger-sized HAp particles at promoting cell proliferation and inhibiting apoptosis of human osteoblast-like MG-63 cells [15]. Although nanoHAp holds immense potential for facilitating enhanced proliferation and differentiation of osteogenic-related cells, crucial for bone regeneration [6], its biocompatibility is still incompletely elucidated [7].

Another notable aspect of nanoHAp is that it serves as an excellent host material, providing remarkable flexibility for functionalization with a wide range of dopants and substrates. To mimic the mineral composition of natural bone and increase the biocompatibility and bioactivity of ceramic implants, researchers have explored various substitutions incorporated within the apatite lattice or adsorbed on the crystal surface [16]. HAp ions can be replaced by isovalent and heterovalent substitutions [17]. These include cationic substitutions, involving the replacement of calcium with monovalent (e.g., Ag^+^, Na^+^, and K^+^), bivalent (e.g., Mg^2+^, Sr^2+^, Zn^2+^, and Ba^2+^), or multivalent cations (e.g., Eu^3+^, Sm^3+^, Ce^3+^, etc.) occupying lattice sites originally held by Ca^2+^ ions, and anionic substitutions, occurring either at hydroxyl (e.g., F^−^ and Cl^−^) or phosphate (e.g., AsO_4_^3−^, SiO_4_^4−^, and CO_3_^2−^) sites or at a combination of both. The presence of these dopants in the structure of nanoHAp results in modifications in the chemical, physical, biological, and mechanical properties [18,19,20].

Important research is dedicated to isovalent cationic substitution of Ca^2+^ ions due to the positive biological effects that have been recorded over time. Mg^2+^ and Sr^2+^ substitutions have been correlated with improved biocompatibility and bioactivity, which promote osseointegrational properties. HAp doped with Zn^2+^ presents promising potential in tooth remineralization, osteoporotic bone reparation, or antibacterial applications [16]. HAp substituted with Co^2+^ can promote collagen fiber synthesis and mineral deposition in bone [21]. Also, heterovalent substitution has been correlated with raised bioactivity. Incorporating K^+^ and Na^+^ into HAp enhances protein adsorption and osteoconduction, respectively [22].

Furthermore, the substitution of Ca^2+^ ions in the HAp structure with foreign ions enhances HAp’s properties, making it a promising candidate for various applications in the biomedical field (such as antimicrobial agents [23], imaging probes [24], drug delivery systems [25], bone tissue engineering [26], etc.). Another study by Kazin et al. reported the successful replacement of OH^−^ groups from the Sr_5_(PO_4_)_3_OH apatite structure with 3d-metal ions, resulting in the formation of linear O-M-O chromophores and yielding a powder displaying a spectrum of colors [27].

The incorporation of lanthanide ions into synthetic HAp as substitutes for calcium is of great interest to researchers developing bone-related biomaterial due to their pronounced capacity to inhibit the bone-resorbing activity of osteoclast-like cells and enhance the osteoblastic differentiation process [28,29]. Among the lanthanides, samarium (^153^Sm) emerges as a pivotal element due to its relative affordability and abundance within the lanthanide group. Its ability to form stable Sm^3+^ cations with a strong affinity for bone minerals, coupled with its antibacterial properties, highlights its importance and makes it an intriguing element for research. ^153^Sm is used in a variety of complexes as a bone-seeking radiopharmaceutical, which relieves the pain associated with bone cancer and provides therapeutic irradiation to osteoplastic bone metastases, including osteosarcomas and bone metastases [30,31,32]. Only a few studies have reported on the incorporation of samarium in HAp. For example, particles of samarium-doped HAp were found to enhance the metabolic activity and proliferation of hFOB 1.19 osteoblast cells and exhibit antimicrobial properties against both Gram-negative and Gram-positive bacteria [33,34]. Composites incorporating samarium-doped P_2_O_5_ glass-reinforced HAp have shown enhanced osteoblastic performance and effective antibacterial activity [35]. Furthermore, ^153^Sm–particulate HAp has been suggested as a potential radiation synovectomy agent in the treatment of chronic synovitis and knee synovitis due to its favorable physical and radiobiological properties [36]. According to the studies previously reported by Zantye et al., the incorporation of Sm^3+^ ions into the inherently fluorescent HAp lattice resulted in enhanced luminescence in the nanoparticles [37]. On the other hand, the newly synthesized SmHAp nanoparticles showed no harmful effects on HeLa cells and were readily absorbed, demonstrating their potential as effective agents for live cell bioimaging [37]. Furthermore, in another study reported by Nabeel, the doping of zinc oxide nanoparticles (ZnO) with samarium (Sm^3+^) led to a considerable increase in the anticancer properties of ZnO [38].

However, the exact mechanism of samarium in bone healing and regeneration is still unclear, and the biocompatibility of HAp nanoparticles is still under debate. More research is needed to fully understand the effects of samarium doping on HAp properties.

In the present study, we synthesized and characterized samarium-doped hydroxyapatite nanoparticles (SmHAp) to investigate whether this lanthanide enhances the biological activity of HAp in terms of viability, proliferation, and differentiation using MC3T3-E1 preosteoblasts as an in vitro model. Additionally, we examined the effects of SmHAp on the wound healing process and the integrity of the F-actin cytoskeleton. Our findings demonstrate that samarium supports the enhancement of HAp biocompatibility and promotes bone tissue recovery in the osteoblast model. This study contributes to the exploration of innovative biomaterials aimed at improving the success rate of bone tissue regeneration.

## 2. Materials and Methods

### 2.1. Samarium-Doped Hydroxyapatite Nanoparticles

The synthesis of Ca_10_(PO_4_)_6_(OH)_2_, (x_Sm_ = 0, Ca/P molar ratio = 1.67, HAp) was performed according to the following equations [39,40,41,42]:

10Ca(NO_3_)_2_ · 4H_2_O + 6(NH_4_)_2_HPO_4_ + 8NH_4_OH → Ca_10_(PO_4_)_6_(OH)_2_ + 20NH_4_NO_3_ + 46H_2_O (1)

Ammonium dihydrogen phosphate was dissolved in 300 mL of deionized water, resulting in a solution with a concentration of 0.5 mol/L (P-solution) [43,44,45]. Similarly, calcium nitrate tetrahydrate was dissolved in 300 mL of deionized water, yielding a solution with a concentration of 1.67 mol/L (Ca-solution) [43,44,45]. The mixed solution of Ca and P underwent continuous stirring for 2 h using a mechanical stirrer. Throughout the reaction, the pH was consistently monitored and maintained at 10 using a solution of NH_4_OH. Finally, the obtained precipitate was washed with deionized water. After the last wash, the precipitate was redispersed in deionized water [43,44,45].

The samarium-doped hydroxyapatite (Ca_10−x_Sm_x_(PO_4_)_6_(OH)_2_, x_Sm_ = 0.2, [Ca + Sm]/P molar ratio was 1.67, SmHAp) nanoparticles were obtained following the procedure described in detail in our previous study [33].

In agreement with previous studies conducted by Alshemary, A.Z. et al. [46], “a different charge compensation mechanism occurs at lower temperatures where a vacancy (□) is created in the HAp structure to maintain charge neutrality [46]”:

3Ca^2+^ ↔ 2Sm^3+^ + □ (vacancy) (2)

As a result, a compound with the formula Ca_10-x_Sm_2x/3□x/3_(PO_4_)_6_(OH)_2_ is obtained [46]. A similar charge compensation mechanism was also proposed by Demirel, B. et al. [47] and Ignjatović, N.L. et al. [48].

The chemical reaction for SmHAp (x_Sm_ = 0.2) is as follows:

(10 − x)Ca(NO_3_)_2_⋅4H_2_O + xSm(NO_3_)_3_⋅6H_2_O + 6(NH_4_)2HPO_4_ + 8NH_4_OH → Ca_10−x_ Sm_x_(PO_4_)_6_(OH)_2_ + 20NH_4_NO_3_ + 46H_2_O (3)

Therefore, a volume of 300 mL of a solution containing Sm(NO_3_)_3_·6H_2_O and Ca(NO_3_)_2_·4H_2_O (Ca and Sm solution) was obtained. Secondly, a volume of 300 mL of a solution containing (NH_4_)_2_HPO_4_ (P solution) was prepared [33]. Then, the mixed solution of Ca, Sm, and P was stirred for 2 h. During the synthesis process of samarium-doped hydroxyapatite nanoparticles, the pH was kept at 10 using a solution of NH_4_OH. At the end, the obtained precipitate was washed with deionized water and centrifugated. After the last wash, the precipitate was redispersed in deionized water.

The suspensions were sterilized through exposure to UVC radiation for 45 min.

### 2.2. X-ray Diffraction (XRD)

Details about the structure of the HAp and SmHAp samples were obtained by using X-ray diffraction (XRD). The measurements were performed with the aid of a Bruker D8 Advance diffractometer with CuKα (λ = 1.5418 Å) radiation (Bruker, Karlsruhe, Germany), equipped with a high-efficiency one-dimensional LynxEye™ 1D linear detector. The data were collected in the 2θ range of 20–70° with a step size of 0.02° and a 5 s time per step.

### 2.3. FTIR Spectroscopy

Firstly, the Fourier-transform infrared spectroscopy (FTIR) data were collected in the 450–3800 cm^−1^ spectral domain using a Spectrum BX spectrometer with 4 cm^−1^ resolution. Then, the second derivative FTIR spectra for both the HAp and SmHAp samples were obtained by using the previously outlined procedure [49].

### 2.4. X-ray Photoelectron Spectroscopy (XPS)

X-ray photoelectron spectroscopy (XPS) investigations were performed using a VG ESCA 3 MK II XPS facility (*E_kα_* = 1486.7 eV), VG Scientific Ltd., East Grinstead, UK). The vacuum analysis chamber pressure was P ~ 3 × 10^−8^ torr. The XPS registered spectrum required an energy window *w* = 20 eV with resolution *R* = 50 eV, with 256 registering channels. The Spectral Data Processor v 2.3 (SDP) software [50] was used to process the XPS spectra.

### 2.5. Transmission Electron Microscopy (TEM)

Information about the morphology of the samples was obtained by using transmission electron microscopy (TEM). The analyses were performed with the aid of a CM 20 (Philips-FEI, Hillsboro, OR, USA) transmission electron microscope with a Lab6 filament (Agar Scientific Ltd., Stansted, UK), operating at 200 kV. Particle size distributions from the TEM micrographs were performed by measuring approximately 500 nanoparticles from different regions of the sample.

### 2.6. Energy-Dispersive X-ray Spectroscopy (EDS)

The energy-dispersive X-ray spectroscopy (EDS) studies were carried out using an FEI Quanta Inspect F microscope (FEI Company, Hillsboro, OR, USA) equipped with an energy-dispersive X-ray device. The 3D representations of the elemental mapping cartographies were also realized using ImageJ software (version 1.53 k) [51].

### 2.7. Cell Culture and Treatment

The MC3T3-E1 preosteoblasts (ATCC CRL-2593, ATCC/LGC Standards GmbH, Wesel, Germany) were cultured in high-glucose DMEM (D2902, Sigma, St. Louis, MO, USA) with 10% fetal bovine serum (10270-106, origin of South America, Gibco, Life Technologies, Carlsbad, CA, USA) and 1% antibiotic–antimycotic mix solution (A5955, Merck, St. Louis, MO, USA) in a humid atmosphere of 5% CO_2_ at 37 °C. When the cells reached 80–90% confluence, 0.25% trypsin and 0.53 mM EDTA were added to detach the cells and produce more cultures or to seed them for further experiments. The MC3T3-E1 cells were treated with SmHAp suspensions at various concentrations (6.25, 12.5, 25, 50, 100, 200, and 400 μg/mL) and incubated for 24 and 72 h. Hap-alone treated and untreated cells were used as controls.

### 2.8. MTT Assay

The colorimetric MTT (3-[4,5-dimethylthiazole-2-yl]-2,5-diphenyltetrazolium bromide) assay is conducted to evaluate cell viability, cell proliferation, and cell metabolic activity based on the reductive activity of nicotinamide adenine dinucleotide phosphate (NADPH)-dependent cellular oxidoreductase enzymes. Here, MC3T3-E1 preosteoblasts were seeded in a 96-well plate at a density of 5 × 10^4^ cells/mL and allowed to adhere overnight. After 24 h and 72 h of exposure to tested suspensions, the media were removed and the cells were incubated with 1 mg/mL MTT solution (M2128, Merck, St. Louis, MO, USA) prepared in phosphate-buffered saline (PBS) at 37 °C for 2 h. For quantification, the MTT–formazan crystals were solubilized in 2-propanol. Absorbance was measured on a FlexStation 3 microplate reader (Molecular Devices, San Jose, CA, USA) at 595 nm and the results are represented as percentages of control (100% metabolic activity). Images of the resulting crystals were acquired using an Olympus IX71 fluorescence microscope (Olympus, Tokyo, Japan).

### 2.9. Trypan Blue Cell Viability Assay

This test differentiates viable from dead cells by using a dye that passes only through the permeabilized membranes of damaged cells. MC3T3-E1 cells were seeded in 24-well plates at a density of 5 × 10^4^ cells/mL. After treatment, the cells were trypsinized and the pellet was resuspended in 100 μL of culture media. Cells were stained by mixing 10 μL of 0.4% Trypan blue solution with 10 μL of cell suspension. The viable cells were counted using a Bürker–Türk chamber.

### 2.10. Lactate Dehydrogenase (LDH) Release Assay

The LDH assay is used to evaluate cytotoxicity and cell membrane integrity. In this study, these were determined by using the Cytotoxicity Detection Kit (LDH), ver. 11, (11644793001, Roche, Basel, Switzerland) according to the manufacturer’s protocol. A volume of 50 μL of culture media from the plate used for the MTT assay was carefully transferred into the corresponding wells of an optically clear 96-well plate. A volume of 50 μL of reaction mixture was added to each well and the plate was incubated for up to 15 min at room temperature in the dark. The absorbance of all samples was measured at 490 nm.

### 2.11. Measurement of NO Production

NO production was assayed as an indicator of an inflammatory response by measuring the nitrite concentration in the supernatant of cultured MC3T3-E1 cells. After treatment, 80 μL of the supernatant was transferred to a 96-well plate and mixed with an equal volume of Griess reagent (1% sulfanilamide, 0.1% naphthyl ethylenediamine dihydrochloride, and 6% H_3_PO_4_). Nitrite concentrations were estimated by measuring the absorbance of the supernatant at 540 nm. A solution of 200 µM sodium nitrite (NaNO_2_) was used to generate a standard curve.

### 2.12. Fluorescent Staining of F-Actin Cytoskeleton

Actin fluorescent staining was performed to display the organization of the F-actin cytoskeleton and the cellular shape. The cells were seeded in 24-well plates at a density of 5 × 10^4^ cells/mL. After treatment, the cells on the substrates were washed twice with PBS, fixed in a 4% paraformaldehyde solution for 20 min, and washed again 2 times with PBS. Then, the cells underwent incubation for 45 min at room temperature under constant agitation with a 0.1% TritonX-100 solution prepared in PBS (with 2% BSA). After washing once with PBS, the cells were incubated with Alexa Fluor 488 phalloidin (A12379, Invitrogen/Life Technologies, Carlsbad, CA, USA) in a 150 nM solution for 45 min at room temperature in the dark. Cell nuclei were stained for 10 min with Hoechst (H3570, Merck, St. Louis, MO, USA, 2 μg/mL solution) after two more washes with PBS and then visualized under an Olympus IX71 fluorescence microscope (Olympus, Tokyo, Japan). Images were acquired and examined using the CellSens Dimension software (version v1.11, Olympus, Tokyo, Japan).

### 2.13. Alkaline Phosphatase Activity

The alkaline phosphatase (ALP) activity assay was performed on days 1 and 3 after treatment. Briefly, the cells were lysed using the UP50H ultrasonicator (Hielscher Ultrasound Technology, Teltow, Germany) at 80% amplitude, for one cycle, three rounds of sonication, 30 s each on ice. A volume of 300 μL of lysate was mixed with 1.7 mL of p-nitrophenyl phosphate (p-NPP) 1.25 mM prepared in Gly-NaOH 0.1 M buffer, pH 10, and incubated for 30 min at 37 °C. After the addition of 5 mL NaOH 0.4 N, the mix was incubated again for 10 min at room temperature. The optical density was read at 405 nm against distilled water. A sample with the same reagents but mixed in a different order was used as a control blank (no ALP activity). The amount of p-nitrophenol (μmoles) produced per min per mL was determined by using the molar extinction coefficient of 18.5 × 10^3^ M^−1^ cm^−1^. The ALP activity (U/mg protein) was obtained by normalizing the data to the total protein content, which was measured by using Bradford reagent (B6916, Merck, St. Louis, MO, USA).

### 2.14. Western Blot Analysis

Western blot analysis was used to measure the relative protein expression levels of ALP. Following treatment, the cells were lysed by means of sonication as described above and the protein content in each sample was quantified by using the Bradford method. Equal amounts (40 μg) of protein samples were separated with 10% SDS-PAGE in Tris-Glycine buffer. The electrophoretic bands were transferred onto a PVDF membrane (IPVH00010, Merck Millipore, Darmstadt, Germany) using a wet transfer system. Blocking and immunodetection were performed using the WesternBreeze Chromogenic Kit, with anti-mouse (WB7103, Invitrogen, Carlsbad, CA, USA), according to the manufacturer’s instructions. The membranes were incubated with primary antibodies against ALP (1:250; mouse monoclonal; sc-137213, Santa Cruz Biotechnology, Dallas, TX, USA) and β-actin (1:1000; mouse monoclonal; A2228, Merck, Darmstadt, Germany) under constant agitation overnight. The immunoblots were scanned with a ChemiDoc Imaging System (Bio-Rad Laboratories, Inc., Hercules, CA, USA) and protein expression was quantified with Image Lab software (ver. 5.2, Bio-Rad, Hercules, CA, USA). β-actin was used as a reference protein to normalize the results.

### 2.15. Wound Healing Assay

This assay was conducted to determine the effects of the tested suspensions on the wound repair process. Cells were cultured in 24-well plates at 1 × 10^5^ cells/well to obtain confluent monolayers. After 24 h, straight wounds were made by using a 200 μL sterile pipette tip and then washed with the medium to remove cell debris. The wound gaps were photographed at different intervals (0, 6, 12, 24, and 30 h) after treatment until the entire cell-free area was covered. For the imaging of cell migration, an Olympus IX73 microscope (Olympus, Tokyo, Japan) equipped with a Hamamatsu ORCA-03G camera (A3472-06, Hamamatsu, Japan) was used. The area (%) of cell-free wounds was measured using ImageJ software (version 1.53 k). Wound closure was calculated using the following formula: wound closure (%) = [(Gap T0 − Gap TΔ)/Gap T0] × 100% (where T0 is the area (%) of the wound measured immediately after scratching and TΔ is the area (%) of the wound measured at 6, 12, 24, and 30 h after the scratch was performed). For statistical analysis, 16 images were examined for each experimental condition.

### 2.16. Statistical Analysis

The results are presented as the mean ± standard deviation of three independent experiments. Two-way ANOVA and Tukey and Dunnett’s multiple comparisons tests were used to analyze the data. The confidence interval used is 95% (0.05) and a value of *p* < 0.05 indicates a statistically significant difference in the sample vs. the control. Statistical analysis was performed using GraphPad Prism (version 8, GraphPad Software, La Jolla, CA, USA).

## 3. Results

### 3.1. X-ray Diffraction (XRD)

**Figure 1:**
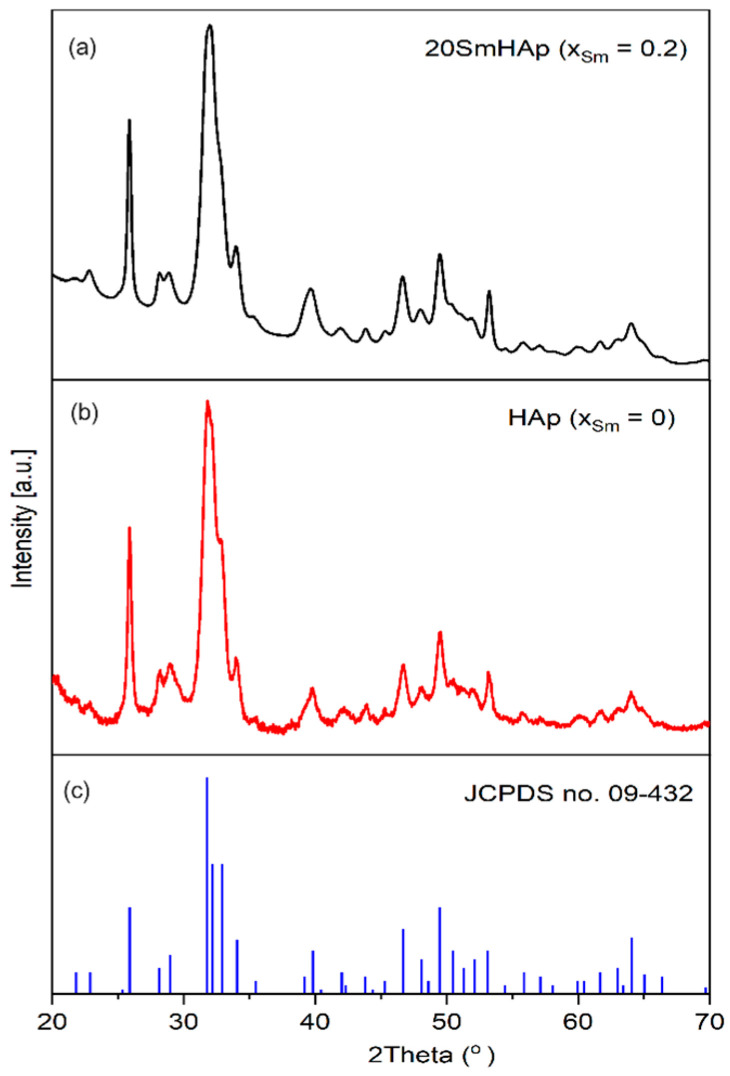
XRD patterns of Ca10(PO4)5 (OH)2 SmHAp (**a**); HAp (**b**) and (JCPDS no. 09-0432) (**c**).

Figure 1 summarizes the XRD patterns of HAp and SmHAp powders obtained by using an adapted co-precipitation method at 100 °C [52]. The reference model for pure hexagonal HAp, JCPDS card no. 09-0432, is also shown in Figure 1. The peaks identified in the SmHAp sample corresponding to the planes (200), (111), (002), (102), (210), (211), (202), (301), (310), (311), (113), (222), (312), (213), and (004) are characteristic of pure HAp according to the card. There was no notable difference in the XRD patterns of both samples regarding the structure. The HAp and SmHAp samples present a stoichiometric pure HAp structure in agreement with JCPDS card no. 09-0432.

No diffraction peaks corresponding to Sm were observed. This fact could be due to the rapid reaction between the phosphate ion and the samarium ion in the solution. However, changes in the SmHAp structure highlight the substitution effect, with a slight shift in the maxima being observed. A slight change was also observed in the lattice parameters and the volume of the unit cell. When part of the Ca^2+^ sites (0.114 nm) is replaced by Sm^3+^ (0.136 nm), the packing of larger atoms tends to distort the lattice parameters.

Since Sm^3+^ has a smaller ionic radius, replacing Ca^2+^ ions with Sm^3+^ ions showed a preference for Ca1 sites. This fact is proven by the values of the lattice parameters and is in agreement with previous studies [53]. The lattice parameters, the volume of the unit cell, and the average crystallite size are presented in Table 1. The ratio of c/a is also presented. A decrease in the c/a ratio of SmHAp suggests a reduction in crystal size according to previous studies [54].

The intensity of XRD characteristic peaks associated with the SmHAp sample decreased compared to the HAp sample. A broadening of the diffraction peaks was also observed for the SmHAp sample. The broadening of the peak and the decrease in intensity in the case of the SmHAp sample could be due to the decrease in particle size and crystallinity as a result of the addition of samarium as a dopant. The Scherrer equation, D = K λ/βcos θ [55], was used to calculate the size of the crystallites, where K is the dimensionless shape factor usually equal to 1, λ is the wavelength of the X-rays, β is the full width at half maximum (FWHM) of the (211) diffraction peak, and θ is the Bragg angle. The crystallite size was 25.4 ± 1 nm for HAp and 21.3 ± 0.89 nm for SmHAp, respectively. The integration of Sm^3+^ ions into the HAp structure led to a decrease in crystallite size. This behavior is in agreement with other results previously obtained [44,56].

### 3.2. FTIR Spectroscopy

**Figure 2:**
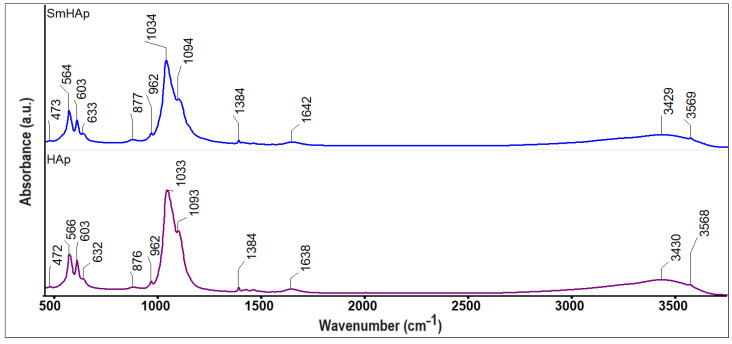
FTIR spectra of HAp and SmHAp samples.

To assess the presence of vibrational bands related to phosphate and hydroxyl groups (specific to the HAp structure) in the obtained samples (HAp and SmHAp), FTIR spectroscopy was employed. In Figure 2, the FTIR spectra obtained for the HAp and SmHAp samples are revealed.

In the following, the FTIR results obtained for the HAp sample will be discussed, which are similar to those obtained for the SmHAp sample. Thus, in Figure 2, the presence of the vibrational bands that indicate the presence of HAp at 472, 566, 603, 632, 962, 1033, 1093, 1638, 3430, and 3568 cm^−1^ can be easily observed. All of these vibrational bands could be attributed to asymmetric and symmetric stretching, as well as to the bending vibrations of phosphate and hydroxyl groups from the HAp structure [33,49,52,57]. The vibrational band observed at 472 cm^−1^ corresponds to the double degenerated bending mode of the O–P–O bond (ν_2_), whereas the vibrational bands noticed between 1030 cm^−1^ and 1100 cm^−1^ are specific to the triply degenerate asymmetric stretching mode of the P–O bond [33,49,52,57]. Furthermore, in the FTIR spectra, one can notice two maxima that are centered at 566 cm^−1^ and 603 cm^−1^ that could be attributed to the ν_4_ vibration of the phosphate group [33,49,52,57]. A vibration band at 632 cm^−1^ is also detected, which originates from the OH^−^ librational mode [33,49,52,57]. The vibration band centered at 962 cm^−1^ is characteristic of the ν_1_ vibration of the phosphate group from the HAp structure [33,49,52,57]. Also, the vibration bands that appear around 1638 cm^−1^ and between 3400 cm^−1^ and 3600 cm^−1^ indicate the presence of the adsorbed water and the hydroxyl group vibration in the analyzed sample [33,49,52,57]. In the SmHAp FTIR spectra, the appearance of additional maxima that could appear due to Sm^3+^ doping of HAp is not observed. More than that, the main difference that can be noticed between the HAp and SmHAp FTIR spectra consists of the fact that the presence of Sm^3+^ induces a broadening and an intensity decrease in the vibrational maxima. According to our previous data, the decrease in the intensity could suggest a decrease in the sample’s crystallinity. These results are in good agreement with the XRD results presented above. Moreover, another effect produced by doping HAp with Sm^3+^ is represented by the slight displacement of the maxima in the case of the SmHAp sample compared with the one of the HAp samples.

**Figure 3:**
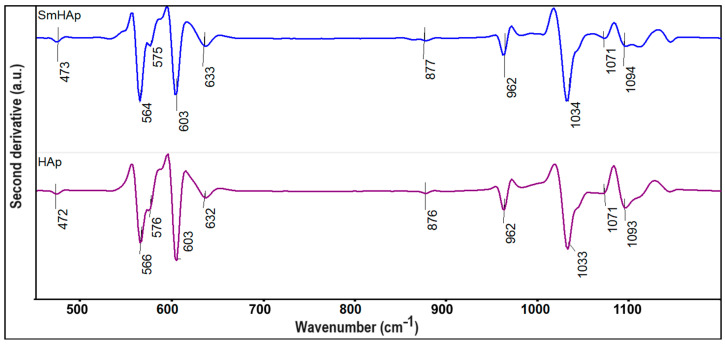
FTIR second derivative spectra of HAp and SmHAp samples.

Furthermore, additional information about the HAp and SmHAp samples was obtained by FTIR second derivative analysis (Figure 3) in the spectral domains that are characteristic of the ν_1_, ν_3,_ and ν_4_ vibrations of the phosphate group from HAp. Moreover, in the analyzed spectral domain, bands that belong to the OH^−^ librational mode at around 632 cm^−1^ can be detected [33,49,52,57]. In Figure 3, a sharp band that is centered at 876 cm^−1^ can be noticed that usually appears due to the B-type substitution of phosphate groups with the carbonate groups. More than that, in the FTIR second derivative spectra, it can be easily observed in another broad band at around 632 cm^−1^, which is specific to hydroxyl groups. The vibration bands observed at 566, 576, and 603 cm^−1^ are specific to the ν_4_ vibration of the PO_4_^3−^ group [33,49,52,57]. On the other hand, in Figure 3, between 1000 and 1100 cm^−1^, maxima that might be attributed to the ν_3_ molecular vibration of the PO_4_^3−^ group from HAp can be found [33,49,52,57]. The intensity of the vibrational bands from the SmHAp second derivative spectra is lower in comparison with the intensity of the maxima of the HAp sample; this behavior is most likely due to the substitution of Ca^2+^ with Sm^3+^ in the HAp structure. Also, the broadening of the maxima underlines a decrease in the degree of crystallinity accompanied by a decrease in the size of the particles. The results reported in this paper are consistent with those previously reported by Iconaru et al. [49] and Ciobanu et al. [33].

### 3.3. X-ray Photoelectron Spectroscopy (XPS)

**Figure 4:**
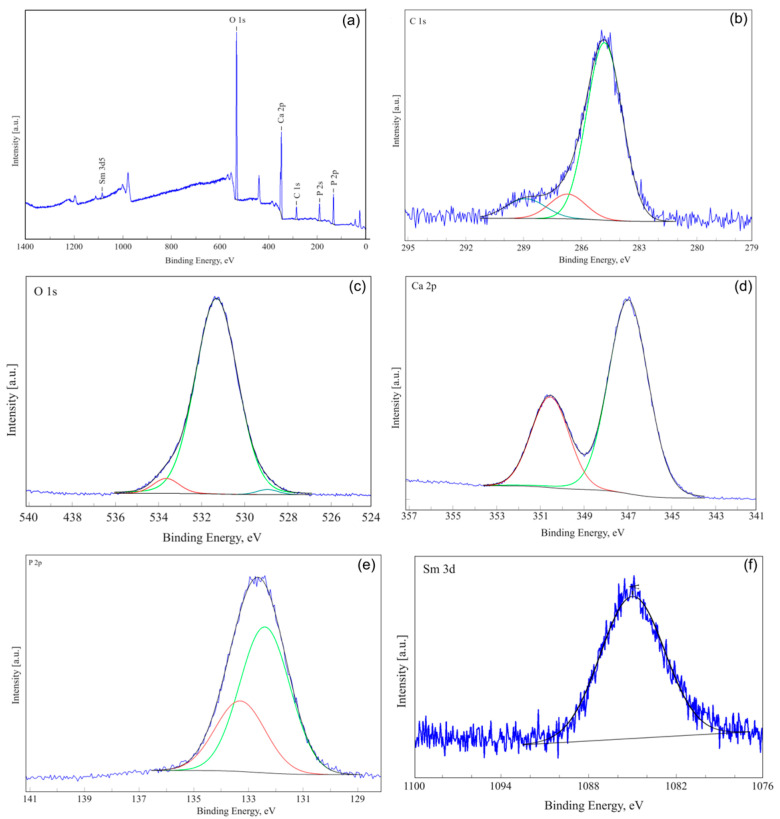
General spectrum (**a**) and high-resolution spectra of C1s (**b**), O1s (**c**), Ca2p (**d**), P2p (**e**), and Sm3d5/2 (**f**) of SmHAp.

In order to obtain qualitative information regarding the presence of samarium in the composition of the SmHAp sample, XPS analysis was performed. Figure 4a shows the general spectrum of SmHAp and the high-resolution spectra of C1s, O1s, Ca2p, P2p, and Sm3d5/2. For XPS analysis, binding energies were calibrated with the C1s reference at 285 eV. The high-resolution XPS spectrum for C1 of the SmHAp sample is shown in Figure 4b. The high-resolution spectrum of C1s shows C-C and C-H single bonds that are observed at a binding energy (B.E) of 284.8 eV [50]. Peaks at approximately 286.7 and 288.89 eV associated with C-O and O=C-O bonds are also observed. The high-resolution XPS spectrum of O1s oxygen for the SmHAp sample is shown in Figure 4c. Three distinct signals are observed at 531.3, 533.6, and 529.2 eV corresponding to Ca-O [58], P-O [59], and O-H [59,60] bonds associated with HAp. In the high-resolution XPS scan of the Ca2p region (Figure 4d), two peaks are noticed (at a binding energy of 347.7 eV and 351.3 eV). The two lines are specific to 2p3/2 and 2p1/2 of the Ca2p doublet [61,62]. High-resolution XPS spectra of phosphorous P2p for the SmHAp sample are shown in Figure 4e. After deconvolution, the two components are observed at a binding energy of 133.3 eV and 132.4 eV which are specific to HAp, in agreement with previous results [63,64]. The peak at 1084.4 eV observed in the SmHAp sample (Figure 4f) is assigned to Sm 3d5/2, consistent with the normal oxidation state of Sm^3+^ [65].

### 3.4. Transmission Electron Microscopy (TEM)

**Figure 5:**
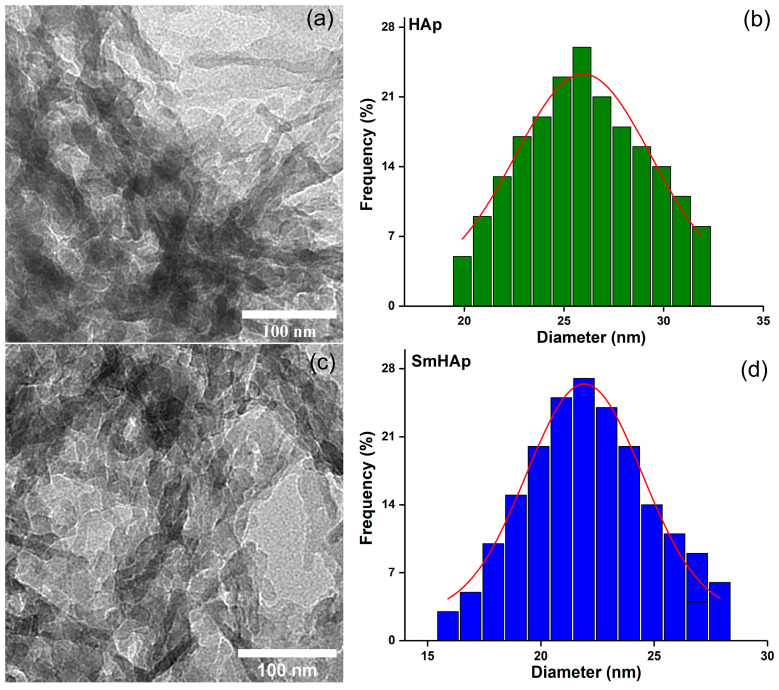
TEM micrographs of HAp (**a**) and HApSm (**c**) and their corresponding particle size distributions (**b**,**d**).

The TEM micrographs of HAp and SmHAp samples are depicted in Figure 5a,c. The TEM micrographs reveal that the samples of SmHAp have a similar shape and maintain the morphology of pure HAp. Both HAp and SmHAp nanoparticles present a uniform ellipsoidal shape, with regular shapes typical of HAp nanoparticles. The particle size distribution obtained from the TEM micrographs depicted in Figure 5b,d highlight that the size of the SmHAp particles is slightly smaller than that obtained for the particles of the HAp sample. The particle size distributions reveal for HAp nanoparticles a distribution size of approximately 25.9 ± 1 nm and a distribution of approximately 21.9 ± 1 nm for the SmHAp samples.

### 3.5. Energy-Dispersive X-ray Spectroscopy (EDS)

**Figure 6:**
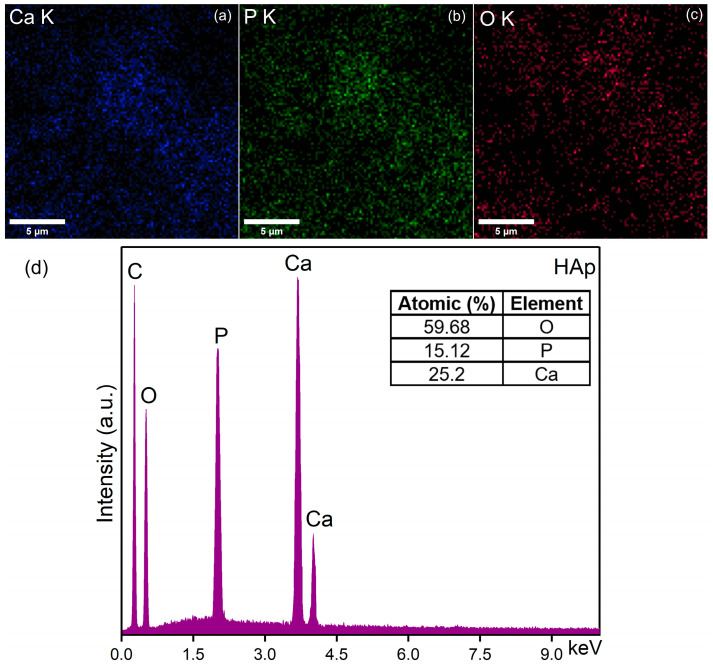
Two-dimensional elemental maps images of HAp sample. (**a**) Ca; (**b**) P; (**c**) O. EDS spectra (**d**).

**Figure 7:**
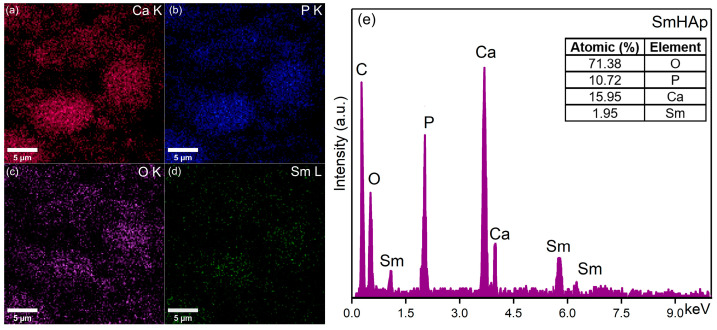
Two-dimensional elemental mapping images of SmHAp sample. (**a**) Ca; (**b**) P; (**c**) O; (**d**) Sm. EDS spectra (**e**).

In Figure 6d and Figure 7e, the results of the EDS qualitative and semi-quantitative analysis obtained for the HAp and SmHAp samples are revealed. Furthermore, the value of the Ca/P ratio obtained for the HAp sample was 1.66 ± 0.1. A similar value was obtained for the (Ca + Sm)/P ratio of the SmHAp sample. Both spectra indicate the samples’ purity and confirm the presence of the main chemical elements from the HAp (Ca, P, and O) and SmHAp (Ca, P, O, and Sm) structures. Also, the distribution of the constituent elements of the SmHAp sample was investigated by using energy-dispersive X-ray spectroscopy (EDS). The EDS mapping of the HAp sample is represented in Figure 6a–c. The results of the EDS mapping reveal the presence of the constituent elements specific to HAp, calcium (Ca), phosphorus (P), and oxygen (O).

The results of the EDS mapping depicted in Figure 7a–d demonstrate the presence of the constituent elements of HAp, calcium (Ca), phosphorus (P), and oxygen (O), as well as the presence of the dopant, samarium (Sm).

**Figure 8:**
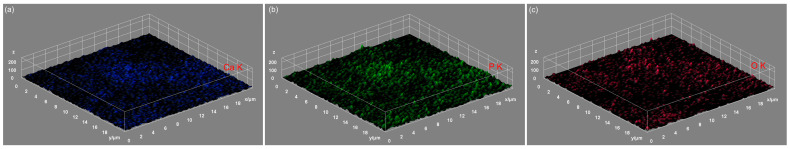
3D Three-dimensional representation of elemental mapping cartographies for HAp sample. (**a**) Ca; (**b**) P; (**c**) O.

**Figure 9:**
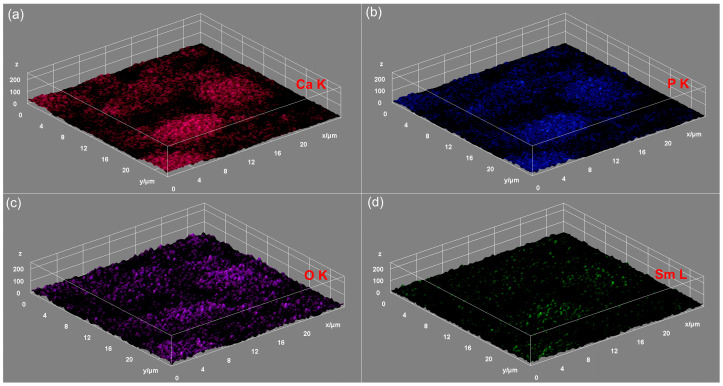
Three-dimensional representation of elemental mapping cartographies obtained for SmHAp sample. (**a**) Ca; (**b**) P; (**c**) O; (**d**) Sm.

The results of the elemental mapping indicate the uniform and homogenous distribution of Ca, O, P, and Sm in the SmHAp sample. Also, the 3D representation of the elemental mapping cartographies obtained for the HAp and SmHAp samples shown in Figure 8a–c and Figure 9a–d confirms the homogenous distribution of the constituent elements in the HAp and SmHAp samples as well as the uniform distribution of the samarium ions in the SmHAp sample.

### 3.6. Osteo-Biocompatibility

Cell viability was quantified by using seven different concentrations (6.25, 12.5, 25, 50, 100, 200, and 400 μg/mL) of both HAp and SmHAp suspensions after 24 and 72 h of incubation in culture media of bone-forming MC3T3-E1 preosteoblasts. Untreated cells were used as a control (100% cell viability). To evaluate the osteo-biocompatibility of our suspensions, we used two cell viability assays: MTT and Trypan blue. The first detects the mitochondrial enzyme activity in viable cells (metabolic active cells) and the second allows us to estimate with approximation the number of both viable and dead cells.

**Figure 10:**
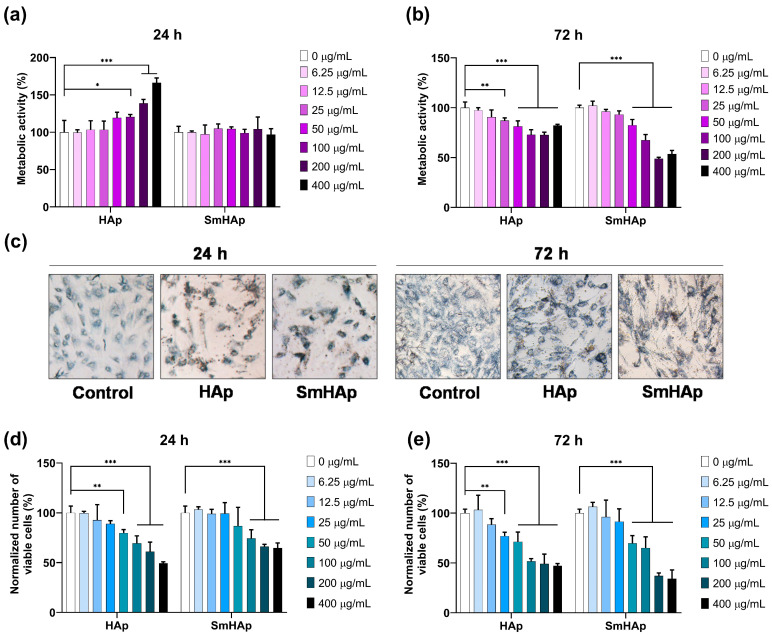
Cell viability of MC3T3-E1 preosteoblasts after exposure to HAp and SmHAp. MTT assay for (**a**) 24 h and (**b**) 72 h; (**c**) microscopy images presenting cells containing formazan crystals (blue–violet) formed after 2 h of incubation with MTT reagent after treatment at concentration of 200 μg/mL; Trypan blue assay after (**d**) 24 h and (**e**) 72 h of exposure. Cell viability was expressed as % of control (control was set at 100%). Statistical differences between untreated and treated groups are calculated using two-way analyses of variance (ANOVA) and results are significant at *p* < 0.05 (*); *p* < 0.01 (**); *p* < 0.001 (***).

Resulting from the MTT data (Figure 10a,b), the SmHAp-treated cells presented a metabolic activity of 97 to 105% compared to the control after 24 h at all concentrations. At 72 h postexposure, the level of cell viability started to decrease below that of the control at concentrations higher than 50 μg/mL (being registered at only 48 to 82% for metabolic activity).

For HAp-treated cells, a significant elevation in mitochondrial enzyme activity at 24 h of incubation with concentrations above 50 μg/mL was observed. Instead, at 72 h postexposure, HAp showed cytotoxicity at high concentrations, starting at 50 μg/mL. Contrary to these results, microscopic examination indicated a reduction in cell density at 24 h. Figure 10c presents the cells after formazan crystals (blue) were formed inside the cytoplasm due to MTT reduction. Those treated with HAp and SmHAp for 24 h are fewer and more intensely colored compared to the control cells. This might suggest that HAp and SmHAp treatments amplify the activity of enzymes responsible for the reduction of the MTT reagent to formazan, thus explaining the increased level of cell viability shown in the MTT data. No interference of HAp and SmHAp suspensions with the MTT reagent was detected in cell-free culture media.

For further clarification, cell viability was also determined using the Trypan blue test. This indicates that the number of viable cells decreased in a dose- and time-dependent manner under HAp and SmHAp treatment. Statistical analysis showed significant differences for the SmHAp sample starting at a concentration of 100 μg/mL at 24 h and 50 μg/mL at 72 h of exposure (Figure 10d,e). For the HAp sample, a cytotoxic effect was observed from a concentration of 50 μg/mL and 25 μg/mL, respectively, for the same intervals of treatment (Figure 10d,e).

### 3.7. Effects on Lactate Dehydrogenase (LDH) Activity and Nitric Oxide (NO) Production

**Figure 11:**
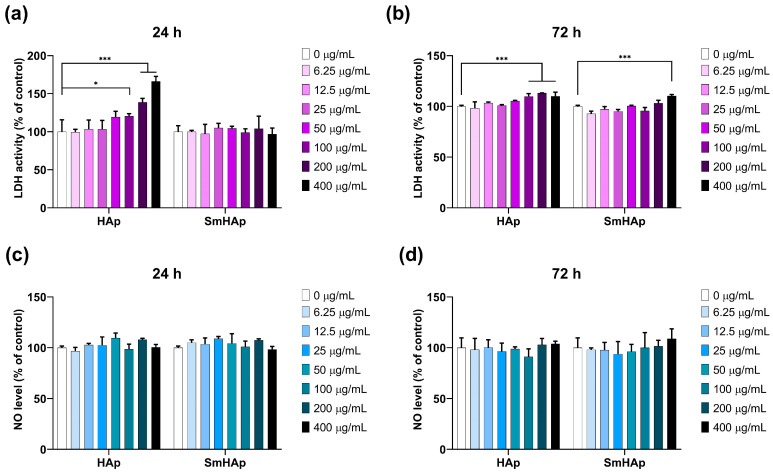
Effects of HAp and SmHAp on cell membrane integrity and NO production of MC3T3-E1 preosteoblasts. LDH activity in culture media after (**a**) 24 h and (**b**) 72 h of incubation with HAp and SmHAp; amount of NO released in culture media in presence of HAp and SmHAp after (**c**) 24 h and (**d**) 72 h. Statistical differences between untreated and treated groups are calculated using two-way analyses of variance (ANOVA) and results are significant at *p* < 0.05 (*); *p* < 0.001 (***).

LDH activity (Figure 11a,b) was quantified in the supernatant of the cells to test the cytotoxicity and membrane integrity. In the control sample, the LDH level was set as 100%. The HAp suspension determined an increase in LDH activity of 13% starting after 24 h at concentrations of 400 μg/mL (*** *p* < 0.001). Moreover, increasing concentrations of HAp from 100 to 400 μg/mL were toxic to MC3T3-E1 pre-osteoblasts after a 72 h incubation, resulting in a loss of membrane integrity and a release of LDH into the surrounding medium at a maximum of 13% compared to the control. As shown in Figure 11a,b, SmHAp treatment did not produce alterations in MC3T3-E1 cell membrane integrity except at the highest dose of 400 μg/mL SmHAp. At this concentration, a slight increase in LDH activity of 10% compared to the control (* *p* < 0.05) was induced after 72 h of incubation.

To assess if the tested materials affect NO production, we measured the nitrite concentration in the culture medium of MC3T3-E1 preosteoblasts. As shown in Figure 11c,d, the treatment of cells with both HAp and SmHAp induced no statistically significant modification in the production of NO for any of the conditions.

### 3.8. Effects on Organization of F-Actin Cytoskeleton

**Figure 12:**
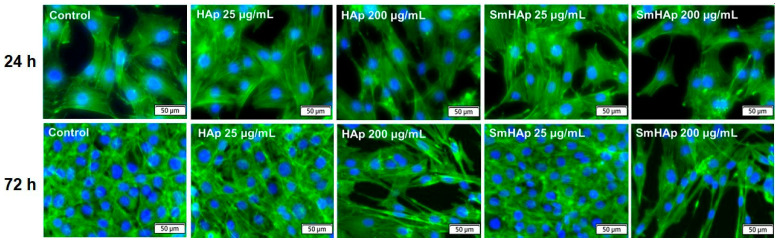
Fluorescent images of the organization of the F-actin cytoskeleton in MC3T3-E1 preosteoblasts exposed to HAp and SmHAp at concentrations of 25 and 200 μg/mL for 24 and 72 h. Actin filaments are stained with Alexa Fluor 488 phalloidin (green) and the cell nuclei with Hoechst (blue). Scale bar, 50 μm.

To evaluate the changes produced at the level of the cellular cytoskeleton after exposure to HAp and SmHAp, fluorescent staining of F-actin filaments was performed. Two concentrations of each nanoparticle suspension were selected in this case, a low one of 25 μg/mL and a higher one of 200 μg/mL. Images showed that treatment with 25 μg/mL HAp or SmHAp did not induce any significant differences in F-actin organization during the experimental period as compared to the control cells. As shown in Figure 12, the control cells have a polygonal shape with smooth borders and are characterized by a dense network of actin stress fibers evenly distributed in the cytoplasm. In contrast, the treatment of MC3T3-E1 cells with 200 μg/mL HAp or SmHAp resulted in strong actin filament elongations, thus losing their specific shape. Moreover, HAp and SmHAp promoted actin filament aggregation in several areas of the cells, which was observed as an intense fluorescent signal in both the cortical and nuclear/perinuclear regions.

### 3.9. Osteoinductive Potential

**Figure 13:**
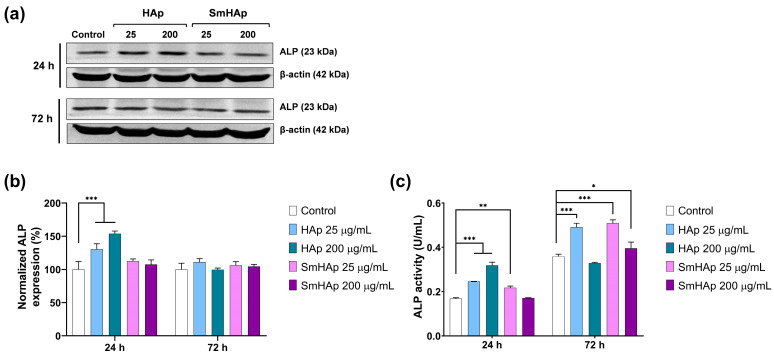
Quantitative results of alkaline phosphatase (ALP) protein expression and enzymatic activity in MC3T3-E1 cells after 24 and 72 h of treatment with HAP and SmHAp. (**a**) Blot images of ALP protein bands; (**b**) level of ALP protein expression normalized to β-actin in cells treated with HAp and SmHAp; (**c**) specific ALP activity in cells treated with HAp and SmHAp expressed as U/mg protein. Statistical differences between untreated and treated groups is calculated using two-way analyses of variance (ANOVA) and results are significant at *p* < 0.05 (*); *p* < 0.01 (**); *p* < 0.001 (***).

The osteoinductive capacity of the HAp and SmHAp suspensions was assessed by examining both the protein expression and enzymatic activity of ALP, an initial marker of osteoblastic differentiation that plays a key role in bone mineralization [66,67]. Following HAp treatment for 24 h with a dose of 25 and 200 μg/mL, ALP protein expression increased significantly by 30% and 54%, respectively, compared to the control level (Figure 13a,b). However, after 72 h, the level of ALP protein expression returned close to the one registered in the control cells. As shown in Figure 13a,b, exposure for 24 and 72 h to SmHAp produced a slight stimulation of ALP protein expression in MC3T3-E1 cells by a maximum of 13% as compared to the control, but statistical significance was not reached in any of the conditions.

Further, ALP enzymatic activity was investigated. This analysis indicated a similar pattern to the one obtained by the Western blot, but the modifications were more pronounced and significant. In the case of cells treated with HAp, a dose of 25 µg/mL was able to significantly stimulate (*** *p* < 0.001) ALP activity by 46% and 37% as compared to the control after 24 and 72 h, respectively (Figure 13c). When the dose of 200 µg/mL was applied, ALP enzymatic activity increased strongly by 89% in the first 24 h and slightly decreased below the control level by 8% after 72 h. Intracellular ALP activity (Figure 13c) in MC3T3-E1 preosteoblasts incubated with SmHAp was elevated by a 25 µg/mL dose by 29% after 24 h and 42% after 72 h as compared to the control cells. The exposure of MC3T3-E1 cells to a higher concentration of 200 µg/mL SmHAp had no significant impact on ALP activity after 24 h. A slight increase of 10% occurred only after 72 h. These results indicate the capacity of HAp and SmHAp to promote bone formation, especially at a dose of 25 µg/mL. At 200 µg/mL, cell osteogenesis is barely activated or even suppressed after 72 h in the case of HAp treatment.

### 3.10. Effects on Preosteoblast Migration

**Figure 14:**
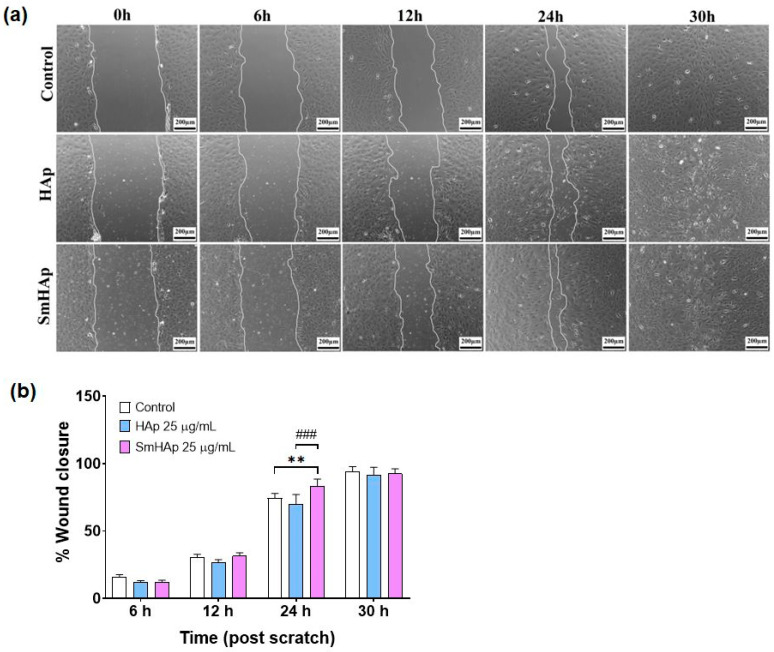
The wound healing assay on MC3T3-E1 preosteoblasts exposed to HAp and SmHAp. (**a**) Representative images (10× magnification) of the in vitro scratch wound healing assay. The outlines show the gap area detected using ImageJ software (version 1.53 k). The scale bar is 200 μm. (**b**) A summary bar graph illustrating the percentages of wound closure at indicated time points during the scratch wound assay. The data (*n* = 16) are presented as percentages relative to the width of the wounds at 0 h (immediately after treatment). Statistical differences between untreated and treated groups are calculated using two-way analyses of variance (ANOVA) and the results are significant at *p* < 0.01 (**); HAp sample vs. SmHAp sample, *p* < 0.001 (###).

To determine the impact of HApSm treatment on MC3T3-E1 cell migration, we performed an in vitro wound healing assay. The concentration used for the treatment of MC3T3-E1 preosteoblasts was 25 μg/mL HAp and SmHAp. The wound was completely closed within 30 h post-scratch, and this was reached almost at the same time for all groups. Our results illustrate that HAp had no significant effect on cell migration of MC3T3-E1 preosteoblasts when compared to the control at subjected intervals of 6, 12, 24, and 30 h (Figure 14a,b). Instead, the assay demonstrated that cell migration into the cell-free region was significantly accelerated by 12% (*p* < 0.01 ***) in the presence of SmHAP when compared to the controls after 24 h (Figure 14a,b). A positive effect of SmHAp on cell migration was also registered in comparison to HAp. The area of the wound was covered with 19% more in the SmHAp group compared with the HAp group after 24 h post-scratch and the results are statistically significant (*p* < 0.001 ^###^).

## 4. Discussion

This is the first study reporting on the biological activity of samarium-doped hydroxyapatite nanoparticles in MC3T3-E1 preosteoblasts. MC3T3-E1 is one of the most commonly used osteoblast-like cell lines to evaluate the biocompatibility of biomaterials and osteoblast differentiation in studies related to bone tissue engineering [68,69]. Osteoblasts are involved in the cycle of bone remodeling by generating the bone matrix [70]. Within bone tissue, osteoblasts that encase the osteoid are responsible for synthesizing HAp [71]. Here, nanoparticles with average particle sizes of 25.4 ± 1 nm for HAp and 21.3 ± 0.89 nm for SmHAp were developed using a cost-effective method (co-precipitation). Their physicochemical characteristics were evaluated through XRD, FTIR, TEM, XPS, and EDS investigations. The main focus of this study was on investigating the biocompatibility and osteogenic activity of SmHAp in comparison with bare HAp.

Previous investigations have shown that HAp nanoparticles can enter cells, with endocytosis being the common mechanism. In liver cancer cells, clathrin-mediated endocytosis was identified as the primary route of HAp nanoparticle uptake [72]. This was also observed in endothelial cells [13] and breast cancer cells [73], where HAp nanoparticles were internalized by means of clathrin- and caveolin-mediated endocytosis, while HAp microparticles were taken up through the micropinocytosis pathway. The internalization of HAp nanoparticles in osteoblasts was found to result in the dissolution and reprecipitation of calcium phosphate within intracellular compartments, suggesting a link to physiological processes controlling intracellular calcium ion concentrations [74]. Similarly, lanthanum-doped HAp nanoparticles were internalized by the human embryonic kidney and adenocarcinoma cells, with the degree of internalization and cytotoxicity being dependent on the concentration of lanthanum [75]. In gastric cancer cells, different HAp nanoparticle preparations, synthesized by using a sonochemistry-assisted microwave method, were internalized through energy-dependent pathways, with the smallest nanoparticles showing the highest uptake efficiency and cytotoxicity [76].

The internalization of HAp nanoparticles may lead to a series of cell responses. Thus, assessing their cytotoxicity is crucial to ensure their safety in biomedical applications. In this study, the cytotoxicity evaluation of HAp and SmHAp nanoparticles on MC3T3-E1 preosteoblasts was achieved by conducting several tests. When the MTT assay was performed, it led to confusing results. In the first 24 h after exposure to HAp, the metabolic activity of the cells increased significantly, starting at a concentration of 50 μg/mL, and decreased after 72 h starting at the same concentration. Exposure to SmHAp produced no modifications after 24 h, but a pronounced decrease in cell viability after 72 h was registered starting at 50 μg/mL. The changes in metabolic viability were not in correlation with the growth inhibition observed after performing the Trypan blue test. While this assay demonstrated increased cytotoxicity for both HAp and SmHAp in a time- and dose-dependent manner, inconsistencies between the results of these two tests could stem from methodological differences. Microscopic observations led us to hypothesize that the formation of MTT–formazan crystals was enhanced after treatment, particularly with HAp. The increase in metabolic activity could be attributed to disturbances in calcium (Ca^2+^) homeostasis following HAp treatment, resulting in mitochondrial hyperactivity [77]. This observation might suggest that these particles may accelerate the enzymatic activity of mitochondrial dehydrogenase, intensifying the reduction of the MTT reagent and leading to erroneously higher absorbance values. Considering these findings, HAp-induced metabolic hyperactivation could limit the utility of the MTT assay as a cytotoxicity test for evaluating HAp particle biocompatibility. This event was less evident in cells treated with SmHAp, suggesting a possible role of Sm^3+^ ions in maintaining a balance of calcium levels. In line with our assumptions, a previous study reported that the accuracy of the MTT test is lower than that of the cell growth inhibition assay when analyzing the cytotoxicity of HAp [78].

The cytotoxic effects of HAp and SmHAp nanoparticles were also evaluated by conducting the LDH assay. More specifically, this test investigates the LDH release from cells with injured membranes following treatment. The results showed that HAp nanoparticles can affect the cell membrane integrity at high doses at both time intervals, which was much more in agreement with the data obtained from the Trypan blue assay. The cytotoxicity evaluation revealed that bare HAp nanoparticles exhibited biocompatibility at relatively low doses with MC3T3-E1 preosteoblasts and inhibited their proliferation at concentrations exceeding 50 μg/mL after 24 h and 25 μg/mL after 72 h. In contrast, SmHAp nanoparticles induced toxic effects exceeding a dose of 100 μg/mL after 24 h and 50 μg/mL after 72 h. Concentrations above 100 μg/mL for HAp and 400 μg/mL for SmHAp could alter membrane permeability. Overall, these findings indicate that Sm doping enhances the biocompatibility of HAp.

Research on the effects of HAp nanoparticles is ongoing within the scientific community, comprising both in vitro and in vivo investigations. The effects are dependent on biological features (cell type and organ), the physicochemical characteristics of the nanoparticles, (chemistry and size), or the experimental set-up (dose and exposure time). However, studies reporting on the cytotoxicity of SmHAp are very limited. They indicate that SmHAp-based materials exhibit low cytotoxicity in certain human cell lines, such as hFOB, HepG2, or HGF-1 [33,79,80].

Nitric oxide (NO) is a small yet multifaceted molecule intricately involved in immune responses and inflammation. During inflammatory processes, it is overproduced in osteoblastic MC3T3-E1 cells by inducible nitric oxide synthase (iNOS) [81]. When generated excessively, NO can trigger a pro-inflammatory cascade or react with superoxide (O_2_^−^) molecules, resulting in the formation of peroxynitrite (ONOO^−^). These peroxynitrite species are powerful oxidants that can further oxidize or nitrate other molecules, leading to the production of other damaging species such as hydroxyl radicals (•OH) and nitrogen dioxide (NO_2_), ultimately contributing to oxidative stress [82]. Our study revealed that treating preosteoblasts with HAp and SmHAp nanoparticles did not induce NO production in any of the conditions. These findings demonstrate a relatively low toxicity associated with the treatment, suggesting that iNOS activation, responsible for NO generation in this context, could not be triggered.

We further explored how HAp and SmHAp treatments influence the actin organization of MC3T3-E1 preosteoblasts. Actin filaments, serving as the dynamic scaffolding within the cellular cytoskeleton, play a pivotal role in osteoblast function during bone formation and remodeling. These bone-forming cells rely on actin for mechanosensing, cell movement, and differentiation [83]. Previously, it was found that actin cytoskeleton reorganization is involved in the cell differentiation of MC3T3-E1 cells [84]. The results of our study showed that at a lower concentration (25 μg/mL), neither HAp nor SmHAp altered F-actin filaments. However, at a higher dose (200 μg/mL), significant changes occurred after 24 and 72 h of exposure. The actin cytoskeleton adopted a distinct arrangement, characterized by perpendicular or slightly oblique actin bundles with wide lamellipodia and long filopodia. These alterations highlight the potential impact of HAp and SmHAp nanoparticles at high doses on actin network organization, which could significantly influence bone formation and architecture by altering cell shape, movement, intracellular cargo transport, adhesion, and cellular interactions even at short exposure intervals. Moreover, the microscopic images displaying F-actin filaments revealed the reduced density of cells at high concentrations, corroborating the growth inhibition observed in the cytotoxicity tests.

Bone formation, also termed extracellular matrix mineralization, is a result of an orchestrated sequence of biochemical events by functional proteins including alkaline phosphatase (ALP) and osteocalcin (Ocn) produced by osteoblasts [84]. The induction of ALP expression has relevance for osteoblast activation, promoting bone matrix formation. This enzyme increases the rate of inorganic phosphates and facilitates mineralization, as well as reducing the extracellular concentration of pyrophosphate, an inhibitor of mineral formation. Mineralization is the production, inside matrix vesicles, of HAp crystals that sprout from the outer membrane of hypertrophic osteoblasts and chondrocytes [71].

To study the induction of ALP by HAp and SmHAp in MC3T3-E1 preosteoblasts, we assessed both protein expression and enzymatic activity. Although the Western blot evaluation was less sensitive compared to the kinetic measurements, both techniques revealed a similar pattern of ALP variation. ALP was significantly induced by HAp and SmHAp nanoparticles at a dose of 25 μg/mL in a time-dependent manner, suggesting their capacity to induce bone formation. According to our results, doping of HAp with Sm^3+^ slightly improved its osteoinductive potential after 72 h. However, a concentration of 200 μg/mL SmHAp slightly increased ALP activity after 72 h, while HAp initially increased ALP activity within the first 24 h and then decreased it after 72 h. This fluctuation might be attributed to alterations in Ca^2+^ homeostasis. Our findings suggest that Sm^3+^ ion substitution modifies the HAp surface, allowing it to interact more favorably with biological molecules and ultimately promoting osteogenesis.

ALP also contributes to the formation of bone tissue during wound healing by creating a supportive matrix for cell migration and tissue regeneration [85]. The HAp and SmHAp nanoparticles were evaluated for wound healing efficiency and the capacity to promote the migration of MC3T3-E1 cells. SmHAp nanoparticles showed a superior wound healing activity compared to bare HAp nanoparticles after 24 h of exposure, which is in correlation with the increased ALP activity. However, cell migration was suppressed after 30 h. The HAp nanoparticles had no effects on MC3T3-E1 cell migration in the tested conditions. These variations in the results might be a consequence of disturbances in Ca^2+^ homeostasis that occurred after 24 h which can alter gene and protein expression patterns, affecting cellular differentiation, proliferation, and migration.

In line with our findings, the impact of Sm doping on the biocompatibility of HAp has also been confirmed in previous studies. For example, the incorporation of Sm^3+^ ions in CaO-P_2_O_5_-based-glass–HAp composites showed improved osteoblastic cell responses, higher cell proliferation, and higher expression of relevant osteoblastic genes [35]. The minimal influence on cell proliferation of samarium–HAp nanoparticles at lower doping concentrations was also demonstrated in HeLa cells [37].

To summarize, this paper presents a preliminary study on the fabrication and characterization of HAp and SmHAp nanoparticle suspensions for biomedical applications. This research study provides valuable insights for designing HAp-based biomaterials essential for bone regeneration and implants. Additionally, the biological results underscore the role of Sm^3+^ ions in enhancing the properties of Hap, influencing osteoblastic cell behavior in various ways. SmHAp positively impacts cell proliferation, influences cell differentiation processes, and plays a role in cell migration, without triggering an inflammatory response. Herein, we showed that Sm^3+^ substitution reduced HAp cytotoxicity in MC3T3-E1 preosteoblasts. The present study also highlights the impact of HAp and SmHAp on actin dynamics and organization in MC3T3-E1 osteoblasts, especially at high concentrations. Furthermore, it suggests the potential effects of HAp and SmHAp on ALP activity and wound healing processes, contributing to bone formation and repair. However, further research is necessary to optimize the samarium concentration in nanoceramic formulations, aiming for enhanced bone regeneration. Additionally, understanding the underlying mechanisms triggered by Sm^3+^ ions in osteoblastic cells will contribute to advancing biomaterial design and bone tissue engineering.

## 5. Conclusions

Hence, our study proposes a substitution with Sm^3+^ ions to enhance Hap’s biological properties in osteo-derived cells. The fabrication of HAp and SmHAp nanoparticles was achieved through co-precipitation. The physicochemical characterization highlighted the presence of HAp in the nanoparticles and depicted the preservation of the HAp structure after the integration of Sm^3+^ ions. The samples presented a nanometric size distribution, with average particle sizes calculated as 25.4 ± 1 nm for HAp and 21.3 ± 0.89 nm for SmHAp, respectively. Furthermore, the EDS mapping analysis reveals the presence of the constituent (Ca, P, O, and Sm) elements and their uniform and homogenous distribution. The results of the in vitro biological assessments indicate that the analyzed HAp and SmHAp nanoparticles are biocompatible with the MC3T3-E1 preosteoblasts’ cell line and sustain their osteogenic activity. Due to physicochemical beneficial characteristics and improved biological compatibility, SmHAp nanoparticles have great potential for being used in future bone tissue engineering applications.

## Figures and Tables

**Table 1:** The lattice parameters, volume of the unit cell, crystallite size, and c/a ratio.

Sample	Lattice Parameter (Å)		c/a Ratio	Volume of Unit Cell (Å)^3^	Crystallite Size (nm)
	a	c			
HAp	9.414	6.882	0.731	528.18	25.4
SmHAp	9.442	6.892	0.729	532.10	21.3

## Data Availability

The data are available upon reasonable request.
